# Successful Live Birth Outcome in A Patient with Empty Follicle Syndrome: A Case Report and Literature Review

**DOI:** 10.1007/s43032-024-01738-x

**Published:** 2024-11-20

**Authors:** Fang Hong, Bin Chen, Liu Liu, Xiaomei Tong

**Affiliations:** 1 Center of Reproductive Medicine, Sir Run Run Shaw Hospital, School of Medicine, Zhejiang University, Hangzhou, China; 2 Key Laboratory of Reproductive Dysfunction Management of Zhejiang Province, Hangzhou, China

**Keywords:** Empty follicle syndrome, Luteinizing hormone/chorionic gonadotropin receptor (LHCGR), Mutation, Ovarian stimulation

## Abstract

Here, we report on a rare case of a successful live birth in a patient with empty follicle syndrome. A 35-year-old woman with ovulatory disorder and a 4-year history of primary infertility conducted in vitro fertilization-embryo transfer (IVF-ET) treatment in our hospital. The patient experienced six controlled ovarian stimulation cycles. In the first two cycles, despite adequate ovarian response, normal development of multiple follicles, and normal serum estradiol (E2) levels, no oocytes were retrieved from these mature follicles during the aspiration procedure. The patient was diagnosed with “empty follicle syndrome”. Whole exome sequencing (WES) identified a missense mutation in the luteinizing hormone/chorionic gonadotropin receptor (LHCGR). In subsequent cycles, we try to increase the trigger dosage, combine gonadotropin-releasing hormone agonists (GnRH agonist) with human chorionic gonadotropin (HCG) for a dual trigger, supplement with luteinizing hormone (LH)-like active substances during the stimulation process, and extend the time between triggering and oocyte retrieval. In the end, successful oocyte retrieval and pregnancy were achieved.

**Supplementary Information:**

The online version contains supplementary material available at 10.1007/s43032-024-01738-x.

## Introduction

Empty follicle syndrome (EFS) is defined as the complete failure to retrieve oocytes with repeated aspiration and flushing of mature ovarian follicles following the induction of ovulation during IVF, despite apparently normal follicular development and estradiol (E2) levels [1]. This syndrome was found to occur in 0.045–7% of patients undergoing IVF treatment [2, 3]. Based on the levels of beta-hCG (β-hCG) on the day of oocyte retrieval, EFS is classified into “false” EFS (FEFS) and “genuine” EFS (GEFS) [4]. FEFS can be remedied by re-administering HCG or GnRH agonists, potentially leading to the retrieval of complete oocytes and favorable pregnancy outcomes. In contrast, GEFS occurs despite high β-hCG levels and optimal β-hCG bioavailability, with neither cumulus-oocyte complexes (COCs) nor oocytes present. The underlying mechanism of GEFS remains obscure. Literature [5] suggests that GEFS may be caused by dysfunctional LH signal transduction with known mutations of LHCGR. In GEFS patients with LHCGR mutations, neither COCs nor oocytes are retrievable. A study by Yariz et al. [6] identified a mutation in the LHCGR gene (p.N400S) in two sisters, which might impair the LHCGR signaling pathway, causing tight adherence of the COCs to the follicle wall. This mutation results in irreversible blockade of the LH signaling pathway, explaining the ineffectiveness of repeated HCG injections in these patients. In EFS cases, 33% are identified as GEFS, while 67% are considered FEFS. We also review the literature about this debated syndrome (Table 1).

**Table 1:** Case reports and series of empty follicle syndrom

Author (year)	No. of patient with FEFS	No. of patient with GEFS	No. of cycles	Final oocyte maturation	Time interval between trigger and OPU (h)	Possible cause	Summary
Elie Snaifer et al. (2008) [7]	1	0	1	r-hCG 250 ug	36	Absense of hCG injection 36h earlier	After a second oocyte retrieval,4 oocytes were retrieved and 2 embryos were transferred. Pregnancy was obtained and patient gave birth to a healthy male baby at term.
Reichman DE et al. (2010) [8]	7	0	2	U hCG 10 000 IU, U hCG 10000IU	36	Low or absent serum levels of hCG	Repeat administration of hCG is not as beneficial as has been described.
Vutyavanich T et al. (2010) [9]	0	1	2	U hCG 10 000 IU	36	A delayed maturation of OCCs in response to HCG	The lower number of immature oocytes in the aspirates and the difficulty in identifying them could lead to a mistaken diagnosis of GEFS.
Smisha M et al. (2011) [1]	0	1	2	U hCG 10 000 IU	36	It could be due to a genetic factor or low bioavailability of hCG administered	GEFS can be managed by using rhCG, rLH, or triggering with the GnRH agonist in an antagonist cycle.
Bentov Y et al. (2012) [10]	0	1	3	U hCG 10 000 IU	36	A novel heterozygous inactivating mutation in exon 1 of the LHCGR gene	A novel mutation may provide a potential genetic mechanism for the poor oocyte recovery in some IVF cases.
R. Beck-Fruchter et al. (2012) [11]	0	1	7	r-hCG*6, GnRH agonist (0.1 mg) 40h+ r-hCG*34h (double trigger)	36/36/36/36/36/36/40	The etiology of GEFS is obscure	Using GnRH agonist for final oocyte maturation and prolonging the interval between ovulation triggering and OPU .
Mitri F et al. (2014) [12]	0	1	2	GnRH agonist (1 mg)	36	Compound heterozgygous inactivating LHCGR mutation	Much is left to discover about the LHCGR and its effects on fertility.
Ping Yuan et al. (2017) [13]	0	1	1	r-hCG, 10 000 IU (hCG)	36/ 36 (left ovary) + 39 (right ovary)	A novel homozygous mutation in LHCGR gene, c.1345G>A(p.Ala449Thr)	The literature describing the relationship between phenotype and genotypes in females is reviewed, and possible aetiologies and treatment options .
Ping Yuan et al. (2019) [14]	0	2	5	U hCG 10 000 IU, U hCG 10 000 IU+GnRH agonist (0.2 mg)	36/36-39/36/36/36	One novel mutation and one known mutation were identified in the ZP1 gene	Our findings provide new evidence for the genetic basis of GEFS and support for the genetic diagnosis of infertile individuals with abnormal oocyte phenotypes.
			2	U hCG 4 000 IU,U hCG 5000 IU	36/36,38,41		
Can Dai et al. (2019) [15]	0	5	2	/	36	Six novel ZP1 mutations	The antral follicles were also defective in normal COC organisation. The lack of ZP1 might lead to oocyte degeneration or increased fragility of the oocyte during follicular puncture, ultimately resulting in EFS.
Xuefeng Lu et al. (2019) [16]	0	3	13	/	/	All affected women harbored pathogenic LHCGR variants	After dual triggering and prolonging the interval between triggering and OPU, all three patients achieved oocytes and high-quality embryos.
			4				
			6				
Zhihua Zhang et al. (2020) [17]	0	3	4	/	/	One homozygous and two compound heterozygous mutations in LHCGR	The mutations caused abnormal LHCGR glycosylation, decreased protein expression, ectopic subcellular localization of LHCGR, and reduced ATP consumption in HeLa cells.
			4				
			2				
Noushin AM et al. (2021) [18]	0	13	/	GnRH agonist (0.1 mg) 40h+ r-hCG36h	/	unknown	Our findings implicate that double trigger and delayed oocyte retrieval is a safe and efficacious treatment strategy for GEFS.
Liwei Sun et al. (2023) [19]	0	3	/	/	/	ZP1 variant results in a premature stop codon, leading to the truncated ZP1 protein. The ZP2 variant, which is situated in the N-terminus, triggers the degradation of a premature termination protein.	These findings expand the mutational spectrum of ZP1, ZP2 and ZP3, and provide new evidence for genetic diagnosis of female infertility.
Yang Xu et al. (2023) [20]	0	1	7	/	36-48	A novel homozygous variant of the LHCGR gene (NM_000233:c.1847C>A)	No down regulation of the pituitary ,high LH levels after triptrelin and HCG triggering and delaying the retrieval time were the critical strategies .

## Case Report

The patient was a 35-year-old with a 4-year history of primary infertility. She had irregular menses since menarche at the age of 13, with a menstrual cycle length ranging from 30 to 60 days. The patient is 170 cm tall, weighs 61 kg, and has a body mass index (BMI) of 21.1. In 2016, she underwent laparoscopic right mesosalpinx cyst resection, bilateral tubal patency, and hysteroscopic polyp removal. In 2018, hysterosalpingography showed that bilateral fallopian tubes were unobstructed. Multiple follicle monitoring in other hospitals indicated poor follicular development. In 2019, the patient visited our hospital for IVF-ET treatment. Her anti-Müllerian hormone (AMH) level was 3.76 ng/ml, and she had a total of 7 antral follicles, 2-5mm in diameter. Her partner's semen analysis was normal, and both had normal chromosomal karyotypes. The patient began IVF-ET in March 2020 for "primary infertility and ovulatory disorder".

### Cycle 1 (Follicular Phase Long Protocol)

The patient was stimulated with 200 IU/day of recombinant FSH β (Puregon, Merck Sharp & Dohme (China) Ltd.), supplemented later with human menopausal gonadotropin (HMG, Zhuhai Lizhu Group Lizhu Pharmaceutical Factory) and recombinant LH (Luveris, Merck Serono Co., Ltd.) due to low LH levels (LH 0.54 IU/L), reaching 1.26 IU/L. On the 8th day, 10,000 IU of HCG (Lizhu Group Lizhu Pharmaceutical Factory) was injected as a trigger. On the day of HCG injection, her E2 was 383 ng/L, the next day 614 ng/L, and β-hCG 190 IU/L. Oocyte retrieval was performed 36 hours later, expecting 5 oocytes. Despite double-lumen needle aspiration of 6 follicles, the follicular fluid was clear, with no granulosa cells or COCs observed. The E2 levels of the patient remained low throughout the cycle, inconsistent with the expected number of follicles, suggesting developmental issues with the follicles. The patient was advised to rest and adjust before undergoing further ovarian stimulation.

### Cycle 2 (Luteal Phase Stimulation Protocol)

The patient started with 187.5 IU/day of HMG. On the 10th day, a trigger of 5,000 IU HCG and 0.1 mg GnRH agonist was administered. On the day of HCG injection, her E2 was 1,201 ng/L, the next day 1,607 ng/L, β-hCG 141 IU/L, and LH 48 IU/L. The E2 levels and post-trigger β-hCG and LH levels were satisfactory this cycle. Oocyte retrieval was scheduled for 36 hours later, expecting 5 oocytes. During the procedure, no oocyte was retrieved,and no granulosa cells were seen from one follicle in the right ovary; no oocytes were obtained from a follicle in the left ovary either, but some granulosa cells were observed. An additional 5000 IU of HCG was immediately administered, and after 6 hours, a second oocyte retrieval was attempted with a double-lumen needle. Despite aspirating 6 more follicles, no oocytes were retrieved.

**Fig. 1:**
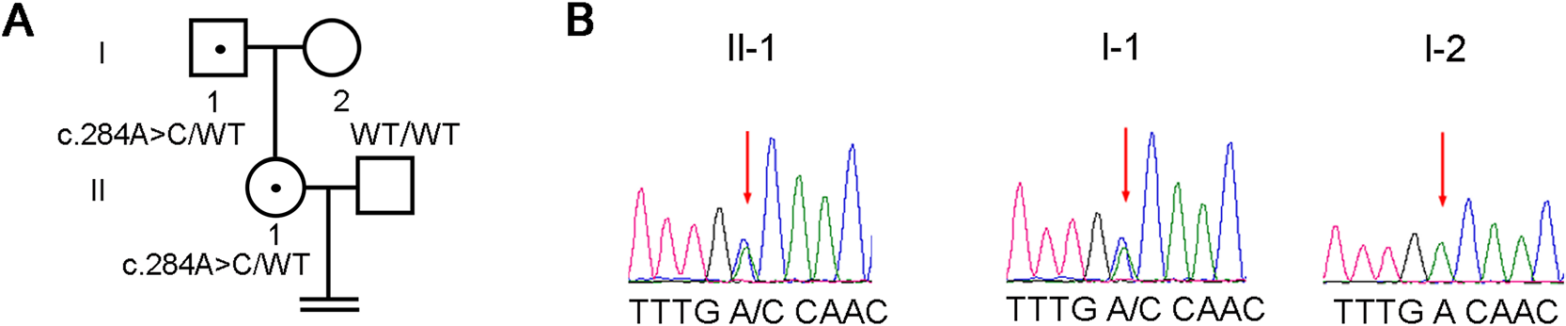
Mutation and sequence analysis of LHCGR. (**A**) Pedigrees of this family ,WT indicates a normal allele , black spot indicates carrier. (**B**) Sanger sequencing chromatograms are shown near the pedigrees. The patient had a single heterozygous missense mutation in the LHCGR gene, inherited from the father

Given the use of GnRH agonist and HCG as trigger medications, and prompt administration of exogenous HCG and a second retrieval attempt 6 hours later (42 hours post-trigger) still yielded no oocytes. WES indicated LHCGR(NM_000233.3): c.284A>C (p.Asp95Ala) mutation, inherited from her father (Fig. 1). This gene variant is associated with LH hormone resistance in females (OMIM: 238320) / AR. According to genetic laws, compound heterozygous or homozygous pathogenic variants of this gene can cause EFS. However, the patient had only a single heterozygous mutation, which cannot explain the failure of repeated oocyte retrievals.

### Cycle 3 (Luteal Phase Long Protocol)

Stimulation was started with 225 IU/day of urofollitropin (Livzon Pharmaceutical, Guangdong, China). On the 8th day, HCG in a dose of 8,000 IU trigger was administered. On the day of HCG injection, her E2 level was 1,211 ng/L, the next day 1,043 ng/L, and β-hCG 174 IU/L. The oocyte retrieval was also conducted 36 hours later, expecting 4 oocytes. Five follicles were aspirated using a double-lumen needle, and one MII oocyte was retrieved. Rescue ICSI was performed, but the embryo culture did not lead to blastocyst formation.

### Cycle 4-6 (Mild Stimulation Protocol)

In cycle 4, the protocol was consisted of Clomiphene Citrate (CC) 1 tablet/day, HMG 150 IU/day, and HCG 200 IU every other day, with the later addition of rLH. On the 9th day, a trigger of HCG 15,000 IU and 0.3 mg GnRH agonist was given in cycle 4. On the day of HCG injection, E2 was 2,141 ng/L, and the next day 2,177 ng/L, with β-hCG at 239 IU/L and LH at 98 IU/L. A bold attempt was made to retrieve oocytes at 40 hours, expecting 7 oocytes. Eight follicles were aspirated, resulting in 4 oocytes, of which 3 were MII and 1 abnormal. One blastocyst was formed and cryopreserved on day 6 and was graded as D6 4CB. In cycle 5-6, the stimulation protocol were similar as that in cycle 4, with normal follicle monitoring and hormone level for the procedure. In cycle 5, a further attempt at 42-hour oocyte retrieval was made. 3 oocytes were retrieved from 6 aspirated follicles, including 2 MII and 1 MI. However, no embryos for transfer in this cycle. In cycle 6, one MII oocyte from two aspirated follicles was retrieved and fertilized via ICSI, resulting in a D6 4CB frozen.

**Fig. 2-7:**
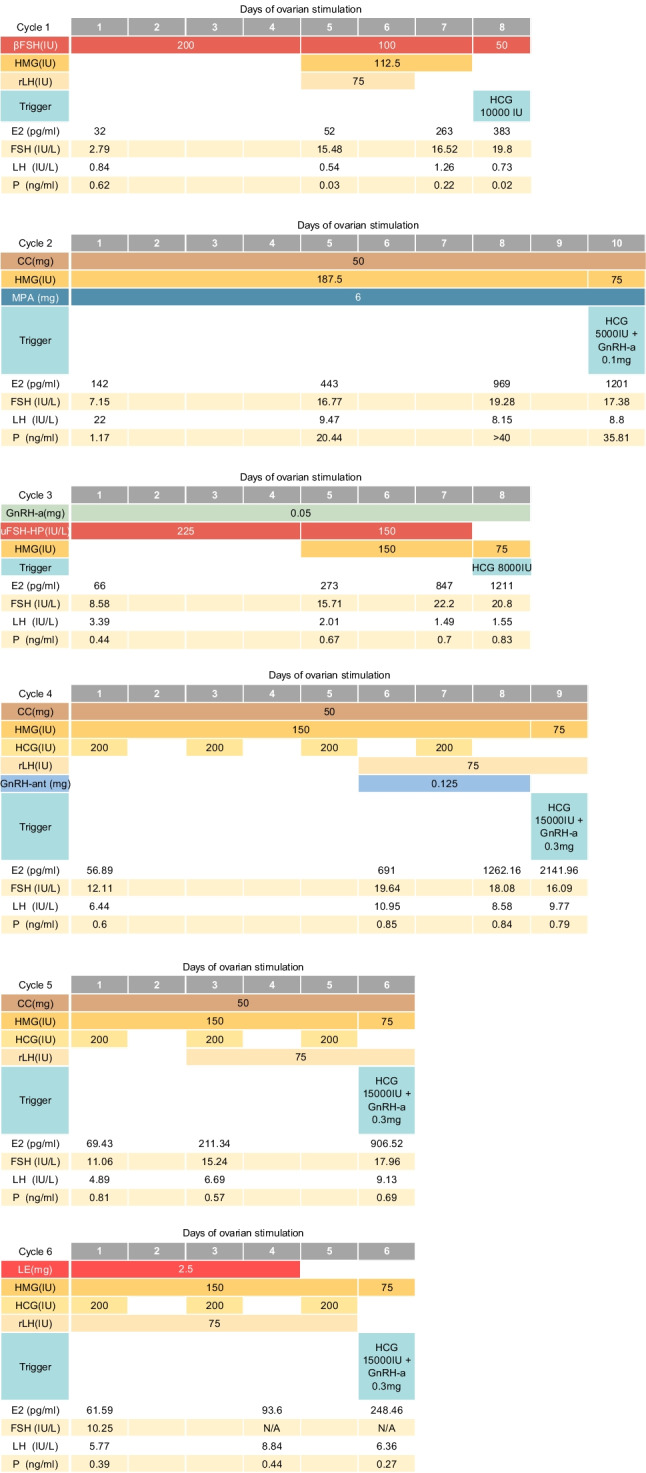
Diagram of ovarian hyperstimulation cycles. βFSH = recombinant follicle-stimulating hormone β; HMG = human menopausal gonadotropin; uFSH-HP = urofollitropin follicle-stimulating hormone, highly purified; rLH = recombinant luteinizing hormone; GnRH-ant = Gonadotropinreleasinghormone antagonist; GnRH-a = Gonadotropin-releasing hormone agonist; LE = Letrozole; hCG = human chorionic gonadotropin; MPA = medroxyprogesterone acetate; E2 = estradiol; FSH = follicle stimulatinghormone; LH = luteinizing hormone; P = Progesterone

Over 6 oocyte retrieval cycles (Figs. 2-7, Table 2) , the patient obtained 2 D6 blastocysts. We used hormone replacement for endometrial preparation, reaching a thickness of 10.5millimetre, type A. Two blastocysts were transferred, resulting in a singleton pregnancy. At 39 weeks of gestation, a healthy infant weighing 4,160g was delivered by cesarean section.

**Table 2:** Details of the stimulation cycles

Cycle	protocol		FSH	rLH	HCG	Gn days	Trigger drug	Time from trigger to egg retrieval	Number of eggs obtained
➀	GnRH agonist	1437.5		150	0	7	HCG10000IU	36	0
➁	Luteal-phase	1687.5		0	0	9	HCG5000IU+ GnRH-a 0.1mg	36	0
➂	GnRH agonist	1800		0	0	7	HCG8000IU	36	1
➃	Microstimulation	1200		300	800	8	HCG15000IU+GnRH-a 0.3mg	40	4
➄	Microstimulation	750		300	600	5	HCG15000IU+ GnRH-a 0.3mg	42	3
➅	Microstimulation	750		375	600	5	HCG15000IU+ GnRH-a 0.3mg	42	1

## Discussion

This case report presents a successful live birth in a patient with EFS. The patient underwent six controlled ovarian stimulation cycles. During the first two cycles, despite adequate stimulation, no oocytes were retrieved, leading to a diagnosis of "empty follicle syndrome". WES revealed a missense mutation in the LHCGR gene, inherited from the father. It belongs to the AR genetic pattern. Theoretically, the phenotype should be normal and there should be no occurrence of EFS. In reality, the patient has repeatedly experienced oocyte retrieval failures. The existing evidence cannot prove that this mutation is the cause of her recurrent empty follicle syndrome phenotype, so the pathogenic mechanism of this patient is unclear. We speculate that there may be the following situations: 1. She also has other trans variants that are outside the scope of WES. 2. This heterozygous mutation has generated a new function that interferes with the normal transcription of LHCGR protein 3. It is not caused by LHCGR variants, but by other genes or non-genetic factors. This case also indirectly proves that WES cannot explain all phenotypes. The human body is complex and there are many non-genetic factors that can affect it.

Although the pathogenic mechanism of our case is unclear, it is sensitive to the treatment of LH addition. We speculate that there may be obstacles in the LH signaling pathway.

Patients with EFS present with an impairment in oocyte maturation. Consequently, we try to increase the trigger dosage of HCG. The β-hCG levels of the patient on the day after trigger in cycle 1-6 were190/141/174/239/252/298 IU/L, respectively. With increasing trigger doses, β-hCG levels showed a dose-dependent increase, enhancing the oocyte retrieval rate. This result was consistent with the findings reported by Abdalla [21]. According to a study by Abdalla, the detachment of the cumulus-oocyte complex and granulosa cells from the follicle wall during ovarian stimulation cycles is dose-dependent on HCG. Successful oocyte retrieval rates for 2,000 IU, 5,000 IU, and 10,000 IU HCG induced ovulation were 77.3%, 99.5%, and 98.1%, respectively. Patients who failed to retrieve oocytes with 2,000 IU HCG succeeded in subsequent cycles with 5,000 IU or 10,000 IU HCG. For this patient, we used a significantly higher trigger dose (HCG 15,000 IU), as three times as the normal dose, and successfully retrieved oocytes.

We also found that oocyte maturation to follicular rupture is a time-dependent process. Different patients require varying amounts of time for the maturation of oocyte-cumulus complexes from exposure to HCG. Some patients may need longer for oocyte detachment from the follicular wall and cumulus expansion [22]. In 2007,Sahebkashaf [23] reported that in 119 EFS cycles, no oocytes were retrieved from 108 patients under standard conditions (36 hours after 10,000 IU HCG injection). However, after waiting an additional 6 hours and re-aspirating the follicles, oocytes were obtained from 85 patients, with an average of 8 ± 4.3 mature oocytes per patient. The intervals between trigger and oocyte retrieval for this patient were 36/36/36/40/42/42 hours respectively, and the oocyte retrieval rate improved with increased intervals.

Modifying the trigger mode can also enhance oocyte maturity and retrieval rates. HCG has long been used as a substitute for the LH surge. Fauser [24] confirmed that GnRH agonist could induce a pre-ovulatory FSH surge, with FSH promoting the formation of LH receptors in luteinized granulosa cells, thus facilitating nuclear maturation of oocytes and cumulus expansion [25]. Lok [26] reported a case where a patient failed to retrieve any oocytes despite high β-hCG levels, and even after switching to a different batch of HCG, oocyte retrieval remained unsuccessful. However, administering 0.2 mg of GnRH agonist as a trigger resulted in the retrieval of nine mature oocytes. For this patient, in subsequent cycles, while increasing the HCG trigger dose, the GnRH agonist trigger dose was also increased (0.3 mg), resulting in successful oocyte retrieval. This might explain why using a dual trigger of HCG and GnRH agonist improves oocyte and embryo quality compared to using HCG alone [27].

For this case, we paid special attention to supplementing LH-active substances. It is known that LH affects granulosa cells and theca cells when the follicular diameter is between 10-12 mm. LH-like substances were added in four out of six stimulation cycles, and in the last three cycles, a combination of HMG, HCG, and rLH was used to supplement LH.HCG, sharing a receptor with LH, acts similarly to LH. Its half-life is longer than LH's and its activity is equivalent to six times that of LH, thus HCG more effectively induces LHCGR receptor expression, providing a more stable stimulated environment [28]. And rLH, directly targets LHCGR. Recent studies have found that rLH and hCG are highly homologous in sequence. Although they act on the same receptor, in vitro experiments show that rLH and hCG have different receptor downstream effects. LH mainly activates the ERK1/2 and AKT pathways, involved in oocyte meiotic division, and promotes granulosa cell proliferation, differentiation, and anti-apoptosis, beneficial for oocyte maturation and thereby improving oocyte and embryo quality [29, 30]. In contrast, HCG primarily activates the cAMP/PKA-mediated steroid synthesis pathway, promoting the synthesis of estrogen and progesterone. HCG's ability to activate cAMP/PKA is five times that of LH [31]. Supplementing LH through multiple pathways improves its binding activity with LHCGR. If there are issues with the binding of LH and LHCGR, follicles can develop but may not mature, potentially leading to EFS. Mutations in LHCGR cause complete or partial loss of response to LH, leading to LH resistance [32–34].

Ultimately, by supplementing LH-like active substances during ovarian stimulation, using a dual trigger of GnRH agonist and HCG, increasing trigger doses, and extending the interval between trigger and oocyte retrieval, we can significantly improve patient prognosis and achieve mature oocytes and healthy live birth. This is consistent with the previously reported treatment strategies for patients with empty follicle syndrome (as shown in Table 1) and once again confirms the effectiveness of these treatments. At the same time, the latest literature also provides some new attempts, such as double trigger (using GnRH agonist 40 h prior to ovum pickup and HCG was added 6 h after the first trigger) [11] and double HCG trigger [35], to improve the prognosis of empty follicle syndrome.

Clinically, encountering EFS should not lead to immediate surrender. Most follicles diagnosed as "empty" in EFS are actually not empty; rather, the oocytes are not aspirated. EFS might represent a syndrome of impaired granulosa cell function, including failure to resume meiotic division in oocytes, inability to expand cumulus, and resistance of immature oocyte-cumulus complexes to follicular stimulation. We also need to leverage genetic testing technology to effectively adjust stimulation protocols and evaluate prognosis.

## Supplementary Information

Below is the link to the electronic supplementary material.

Supplementary file1 (DOCX 883 KB)

## Data Availability

The data supporting the results of this article are included within the article.

## Declarations

The datasets generated during and/or analyzed during the current study are available from the corresponding author on reasonable request.
