# Supplementary material for: Successful Live Birth Outcome in A Patient with Empty Follicle Syndrome: A Case Report and Literature Review
Source: Reprod Sci. 2024 Nov 20;32(3):660–7. doi: 10.1007/s43032-024-01738-x (PMC11870976; doi:10.1007/s43032-024-01738-x)
Supplement: Supplementary file 1 — Supplementary file1 (DOCX 883 KB) [file 43032_2024_1738_MOESM1_ESM.docx]

**Successful Live Birth Outcome in A Patient with Empty Follicle Syndrome：**

**A Case Report and Literature Review**

Figure 1


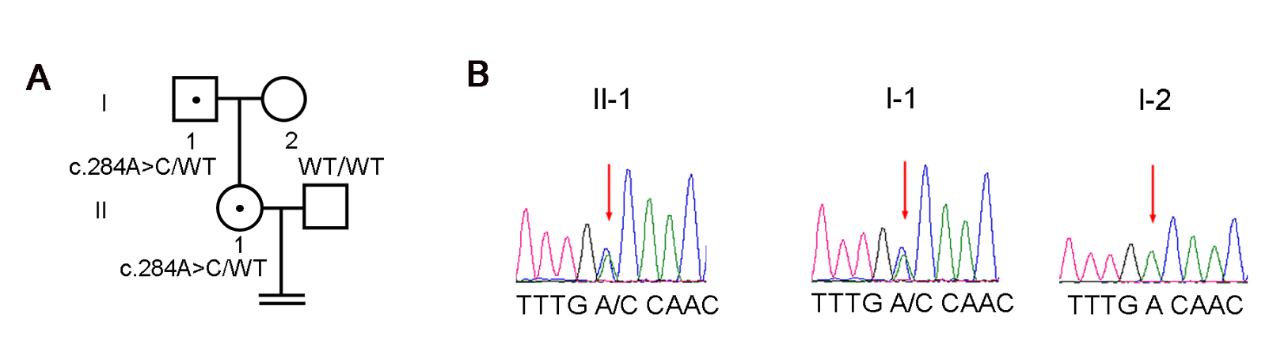


Figure 1 Mutation and sequence analysis of *LHCGR*. (A) Pedigrees of this family ,WT indicates a normal allele , black spot indicates carrier. (B)Sanger sequencing chromatograms are shown near the pedigrees. The patient had a single heterozygous missense mutation in the *LHCGR* gene, inherited from the father.

Figure 2-7 Cycle1-6


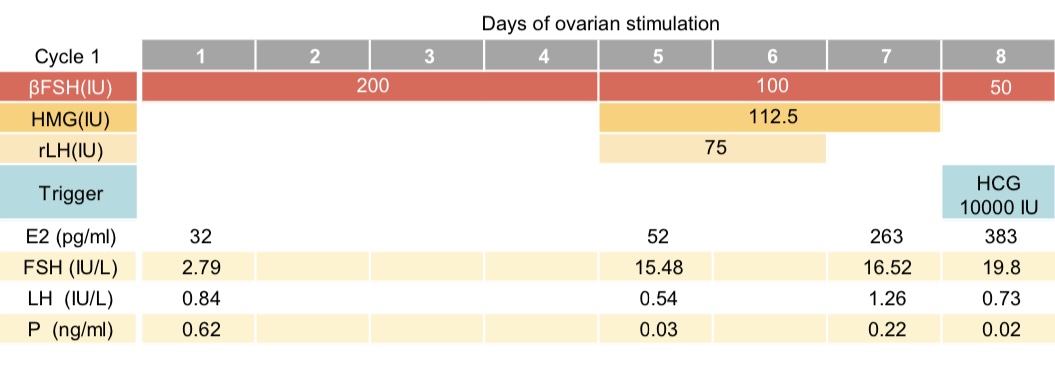


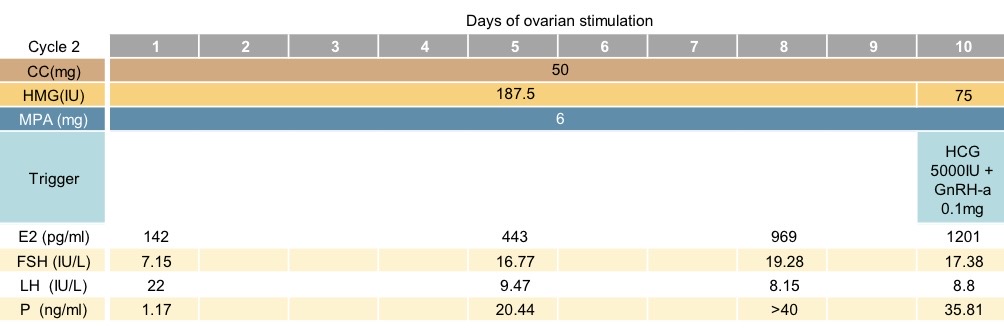


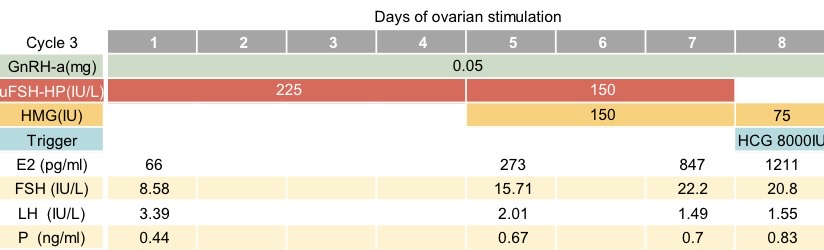


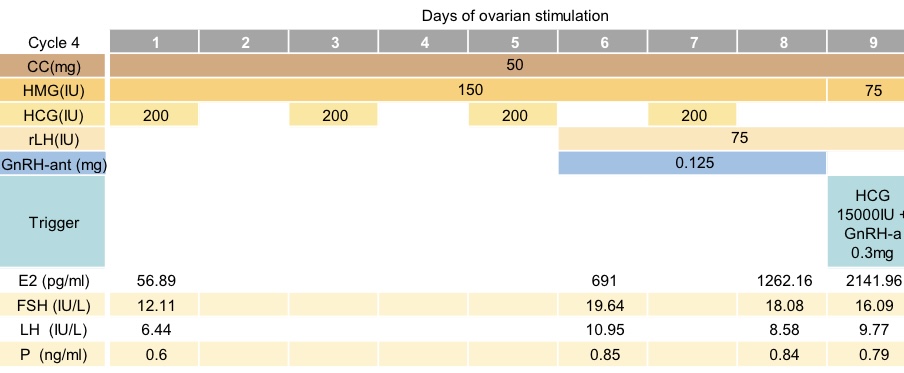


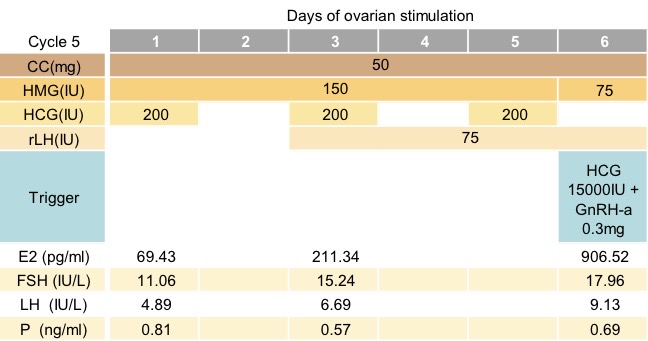


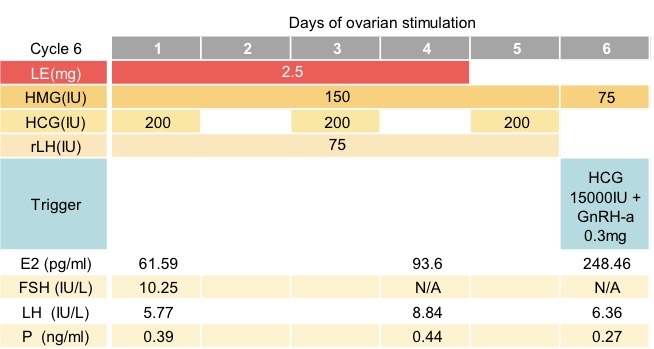


Figure 2-7 Diagram of ovarian hyperstimulation cycles. βFSH = recombinant follicle-stimulating hormone β; HMG = human menopausal gonadotropin; uFSH-HP=urofollitropin follicle-stimulating hormone, highly purified；rLH= recombinant luteinizing hormone ;GnRH-ant= Gonadotropin-releasinghormone antagonist; GnRH-a=Gonadotropin-releasing hormone agonist; LE=Letrozole; hCG = human chorionic gonadotropin; MPA =medroxyprogesterone acetate; E2=estradiol; FSH=follicle stimulatinghormone; LH=luteinizing hormone; P=Progesterone;

Table 1 Case reports and series of empty follicle syndrome


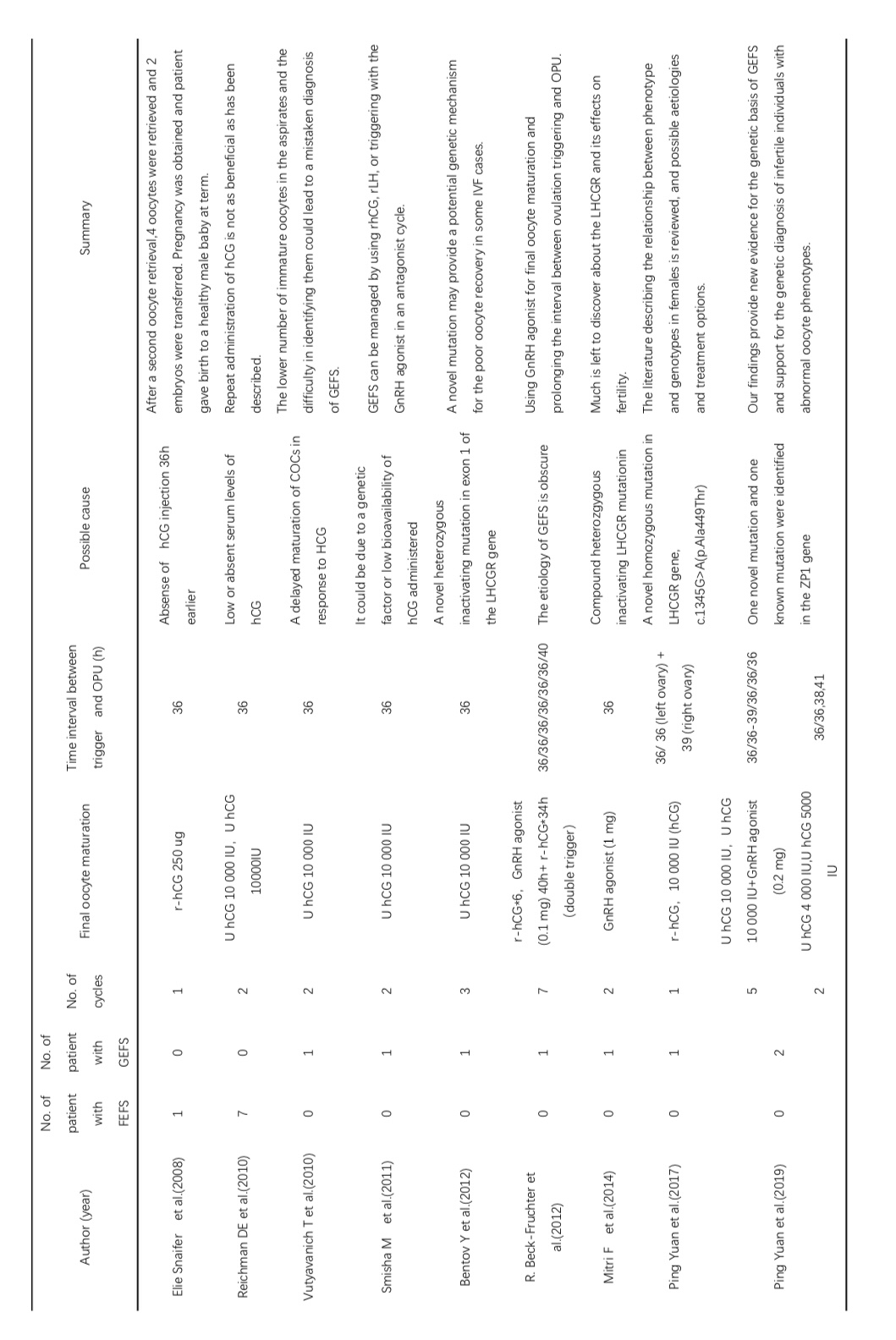


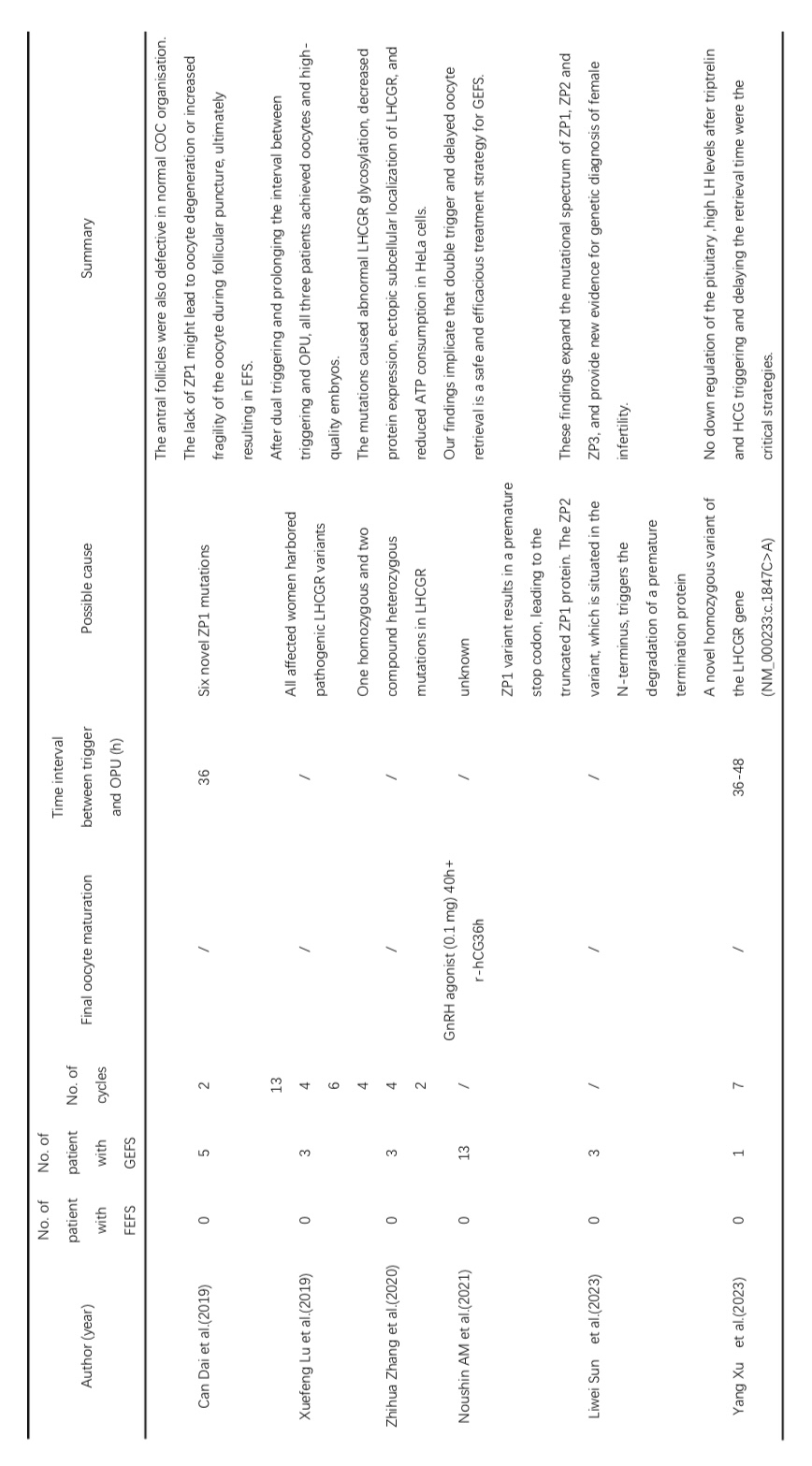


OPU：ovum pick up

Table 2 Details of the stimulation cycles

| Cycle | protocol | | FSH | rLH | HCG | Gn days | Trigger drug | Time from trigger to egg retrieval | Number of eggs obtained |
| --- | --- | --- | --- | --- | --- | --- | --- | --- | --- |
|  | GnRH agonist | 1437.5 | | 150 | 0 | 7 | HCG10000IU | 36 | 0 |
|  | Luteal-phase | 1687.5 | | 0 | 0 | 9 | HCG5000IU+  GnRHa0.1mg | 36 | 0 |
|  | GnRH agonist | 1800 | | 0 | 0 | 7 | HCG8000IU | 36 | 1 |
|  | Microstimulation | 1200 | | 300 | 800 | 8 | HCG15000IU+GnRHa0.3mg | 40 | 4 |
|  | Microstimulation | 750 | | 300 | 600 | 5 | HCG15000IU+  GnRHa0.3mg | 42 | 3 |
|  | Microstimulation | 750 | | 375 | 600 | 5 | HCG15000IU+  GnRHa0.3mg | 42 | 1 |
|  |  |  | |  |  |  |  |  |  |

FSH= follicle-stimulating hormone ；rLH= recombinant luteinizing hormone ；HCG = human chorionic gonadotropin
